# Conservation Assessment of the State Goat Farms by Using SNP Genotyping Data

**DOI:** 10.3390/genes11060652

**Published:** 2020-06-13

**Authors:** Rabiul Islam, Zhangfa Liu, Yefang Li, Lin Jiang, Yuehui Ma

**Affiliations:** 1 National Germplasm Center of Domestic Animal Resources, Institute of Animal Science (IAS), Chinese Academy of Agricultural Sciences (CAAS), No. 2 Yuanmingyuan West Rd., Haidian, Beijing 100193, China; md.rabiul27@yahoo.com (R.I.); yefanglee1994@163.com (Y.L.); jianglin@caas.cn (L.J.); 2 Department of Livestock Services of Ministry of Fisheries and Livestock, Farmgate, Dhaka 1215, Bangladesh; 3 The National conservation farm of Ningxia Zhongwei goats, Zhongwei, Ningxia 755006, China; Zhangfa@yahoo.com

**Keywords:** SNP, *N_e_*, inbreeding coefficient, genome coverage, runs of homozygosity

## Abstract

Conservation of genetic resources is of great concern globally to maintain genetic diversity for sustainable food security. Comprehensive identification of the breed composition, estimation of inbreeding and effective population size are essential for the effective management of farm animal genetic resources and to prevent the animals from genetic erosion. The Zhongwei male (ZWM), Arbas Cashmere male (ACM) and Jining Grey male (JGM) goats are conserved in three different state goat farms in China but their family information, level of inbreeding and effective population size are unknown. We investigated the genomic relationship, inbreeding coefficient and effective population size in these three breeds from three state goat farms using the Illumina goat SNP50 BeadChip. Genomic relationships and phylogenetic analysis revealed that the breeds are clearly separated and formed separate clusters based on their genetic relationship. We obtained a high proportion of informative SNPs, ranging from 91.8% in the Arbas Cashmere male to 96.2% in the Jining Grey male goat breeds with an average mean of 96.8%. Inbreeding, as measured by *F_ROH_*, ranged from 1.79% in ZWM to 8.62% in ACM goat populations. High *F_ROH_* values, elevated genomic coverage of very long ROH (>30 Mb) and severe decline in effective population size were recorded in ACM goat farm. The existence of a high correlation between *F_HOM_* and *F_ROH_* indicates that *F_ROH_* can be used as an alternative to inbreeding estimates in the absence of pedigree records. The *N_e_* estimates 13 generations ago were 166, 69 and 79 for ZWM, ACM and JGM goat farm, respectively indicating that these goat breeds were strongly affected by selection pressure or genetic drift. This study provides insight into the genomic relationship, levels of inbreeding and effective population size in the studied goat populations conserved in the state goat farms which will be valuable in prioritizing populations for conservation and for developing suitable management practices for further genetic improvement of these Chinese male goats.

## 1. Introduction

In order to preserve animal genetic resources and promote sustainable livestock improvement in changing environmental conditions and demands, it is critical to assess the conservation status of genetic resources. Approximately 30% of the world’s farm animal breeds are at risk of extinction due to a lack of conservation policies [1]. Conservation policies of native goat breeds largely depend on the extent of knowledge about history and degree of genetic relationships within and across breeds, inbreeding status of a population, effective population size and characterization of breeds. For accurate assessment of conservation parameters, reliable pedigree data is required. However, pedigree data is difficult to record or sometimes unavailable due to error in recording and might not reflect the real relationship. Molecular data, in particular, single nucleotide polymorphism (SNP) markers are more effective in the assessment of breed composition than pedigree data [2].

Inbreeding is of concern in the livestock industry as it has negative effects on the performance and fitness of animals. The increase in inbreeding in the livestock population tends to have higher rates of congenital disorders and may reduce the survival rates, fertility and long term viability known as inbreeding depression [3]. The rates of inbreeding increase with the increasing use of genetic selection focusing on certain characters. Inbreeding, generally expressed by the inbreeding coefficient (*F*), is the probability of having pair of alleles at a locus that are identical by descent (IBD) with respect to the base population in which all the alleles are assumed unrelated [4]. The inbreeding coefficient is a key parameter to estimate the amount of mating between related individuals that have taken place in a population and is traditionally calculated from pedigree information. However, in practice, pedigree information has some limitations: (i) difficult to obtain, (ii) potentially not reliable due to errors in recording, and (iii) can only be recorded for limited generations [5,6,7].

Advances in high throughput genotyping, sequencing and bioinformatic analysis have facilitated the assessment of inbreeding based on single nucleotide polymorphisms (SNPs) as an alternative to the traditional pedigree-based inbreeding coefficients. In recent times there is a growing interest to estimate the inbreeding coefficient from SNP genotyping data because of its easy estimation, accuracy and ability to trace back to many generations in the past in assessing inbreeding [8]. Moreover, molecular genetics has opened the new opportunity to identify long stretches of homozygous genotypes at individual and population levels, commonly known as runs of homozygosity (ROH). The extent of ROH can also be used to estimate the inbreeding coefficient [9,10]. The longer the ROH, the more likely that recent inbreeding occurred within a pedigree, whereas a shorter one indicates more ancient family relatedness [11]. The proportion of the genome covered by ROH can also be used to calculate the inbreeding coefficient which has shown to be more accurate than the inbreeding coefficient estimated from pedigree data [4].

Effective population size (*N_e_*) is widely regarded as the most important parameter in conservation genetic because of its relationship to inbreeding, selection, migration and mutations [12]. It also helps to us discover the demographic history of a population and allows us to quantify how a particular population will be affected by genetic drift or inbreeding [13]. A smaller *N_e_* increases the chance of inbreeding and genetic drift and decreases the genetic diversity, which may compromise the genetic viability of a population and change the patterns of ROH in the long term [14]. The Arbas Cashmere goat breed with the largest population size is expected to have the greatest diversity and least inbreeding level whereas the opposite effects are expected for the Zhongwei goats with the smallest population size.

China has a total of 193 state farms and conserved 159 livestock breeds. Usually, each breed has been kept in at least one state farm. The state goat farm keeps at least six different pedigrees, 25 males and 250 females. The Zhongwei male (ZWM), Arbas Cashmere male (ACM) and Jining Grey male (JGM) goats are conserved in three different state goat farms in China but their pedigree information, level of inbreeding and effective population size are unknown. The scope of this study was to ensure that the conservation goat farms have at least six unrelated groups of animals, a low level of inbreeding and a sufficiently large number of effective population sizes based on an accurate estimation by the SNP genotyping data.

## 2. Materials and Methods

### 2.1. Ethics Statement

Ethical approval for animal survival was provided by the animal ethics committee of the Institute of Animal Science, Chinese Academy of Agricultural Sciences (IAS-CAAS) (Number IASCAAS-AE-03).

### 2.2. Sampling, Genotyping and Quality Control

A total of 98 male goat individuals comprising of Zhongwei male, Arbas cashmere male, Jining Grey male goats from the three Chinese state goat farms were genotyped using the Illumina Goat SNP50K Beadchip panel (Illumina, San Diego, CA, USA). The detailed sample descriptions are shown in Table 1.

Quality control of the SNP data was implemented using Plink 1.07 [15]. An SNP was filtered from the panel if the following thresholds were not attained: (i) SNP call rate greater than 95%, (ii) an SNP minor allele frequency greater than 0.05, (iii) an SNP with a genotyping rate greater than 95%, (iv) a maximum individual missing genotype rate of more than 10%, or (5) Hardy–Weinberg equilibrium > 1 × 10^−5^.

### 2.3. Genomic Relationship Analysis, and Population Structure

The genomic relationship matrix (G) is an estimator of the actual proportion of the genome that is identical by descent across individuals. The genomic relationship matrix (G) was calculated using G matrix software based on the method proposed by VanRaden [16]. The formula is as follows:

(1) $$G=\frac{ZZ^{\prime }}{2\sum P_{i}\left(1-P_{i}\right)}$$

where *P_i_* is the frequency of the *i*th allele. The genomic inbreeding coefficient for individual j is simply G_jj_ − 1, and genomic relationships between individuals j and the individual k, are obtained by dividing elements G_jk_ by square roots of diagonals G_jj_ and G_kk_.

To further confirm the phylogenetic relationships among the individuals of each breed, we constructed a neighbor-joining (NJ) tree using MEGA 7.0 [17]. We calculated the genetic distance matrix using Plink 1.90 [15] (parameter: distance-matrix) and then, the tree was visualized using MEGA software.

To assess the genetic relatedness between individuals, a principal component analysis (PCA) was performed with Plink 1.90 [15]. To evaluate fine scale genetic sub-structure and determine whether the three male goat breeds represent distinct genetic units, admixture and structure were assessed with Admixture 1.2 [18].

The proportion of SNPs that displayed polymorphic (*P_N_*) was measured as the fraction of total SNPs with MAF greater than 1% in each breed using the PLINK 1.90 software. The average heterozygosity was calculated with the command --hardy using the same software.

### 2.4. Runs of Homozygosity Calling Option and Estimation of Inbreeding

The inbreeding coefficient based on ROH *(F_ROH_*) was estimated as the proportion of genome in ROH over the overall length of the genome covered by the involved SNPs using detectRUNS packages in R software [19]. The following parameters were set to define the ROHs: (i) a minimum length of ROH was set to 1 Mb, (ii) the maximum distance between two consecutive SNPs was allowed to 1 Mb, (iii) one possible heterozygote and one missing genotype were allowed for each ROH, and (iv) to minimize the number of ROH that could occur by chance, the minimum number of SNPs that constituted an ROH was calculated by the formula proposed by Lencz, Lambert [20]:

(2) $$l=\frac{\ln\alpha /\left(n_{s}\times n_{i}\right)}{\ln\left(1-het\right)}$$

where α is the false positive ROH in percentage (set to 0.05 in this study), *n_s_* is the number of SNPs per individual, *n_i_* is the number of individuals tested, and *het* is the proportion of heterozygosity across all SNPs.

The genomic inbreeding coefficient (*F_ROH_*) for each breed was calculated using the following method [21]:

(3) $$\;\;\;\;\;F_{ROH}=\frac{\left(L_{ROH}\right)}{\left(L_{AUTO}\right)}$$

where *L_ROH_* is the total length of ROH of each individual in the genome and *L_AUTO_* is the length of the autosomal genome of the goat (2399.4 Mb). In addition, the chromosomal *F_ROH_* values were also estimated for each breed as summing the total length of an individual’s ROH in each chromosome divided by the length of each chromosome covered by the involved SNPs [22].

To test the fairness of *F_ROH_*, we also calculated the inbreeding coefficient as a function of the expected and observed numbers of homozygous difference (*F_HOM_*) with the following equation:

(4) $$F_{HOM}=\frac{\left(E_{HOM}-O_{HOM}\right)}{\left(L-E_{HOM}\right)}$$

where *L* is the number of autosomal SNPs genotype, *E_HOM_* and *O_HOM_* are the number of expected and observed homozygous genotypes, respectively.

The correlation between *F_ROH_* and *F_HOM_* was calculated across the breeds.

The distribution of ROH coverage can be used to infer the recent and ancient inbreeding. Therefore, we also calculated the average ROH length for four length categories (0–5, 5–15, 15–30, and >30 Mb) to see the distribution of recent and ancestral inbreeding. The average ROH length for each class of each breed was estimated by adding up all ROH segments of each ROH length class per breed and dividing by the total number of individuals of that respective breed.

### 2.5. Effective Population Size (N_e_)

The effective population size (*N_e_*) was calculated for each breed separately using SNeP v1.1 [23]. *N_e_* estimates at different time points are based on the extent of linkage disequilibrium, using the formula proposed by Sved [24]:

(5) $$E\left(r^{2}\right)=\frac{1}{1+4N_{e}C}$$

where *N_e_* is the effective population size, *c* is the genetic distance in Morgans between two markers, and *E*(*r*^2^) is the expected average coefficient of determination (*r*^2^) value for distance *c*. Each genetic distance (*c*) corresponds to a time point value of *t* generation in the past obtained as described by Hayes, Visscher [25]:

(6) $$T=\frac{1}{2c}$$

## 3. Results

### 3.1. Sample and SNP Filtration

In this study, 53,347 SNPs were considered before quality control. After quality control, a common subset of 45,041 SNPs from 98 individuals was used for downstream analysis (Table 2).

### 3.2. Genomic Relationship Analysis, and Population Structure

**Figure 1:**
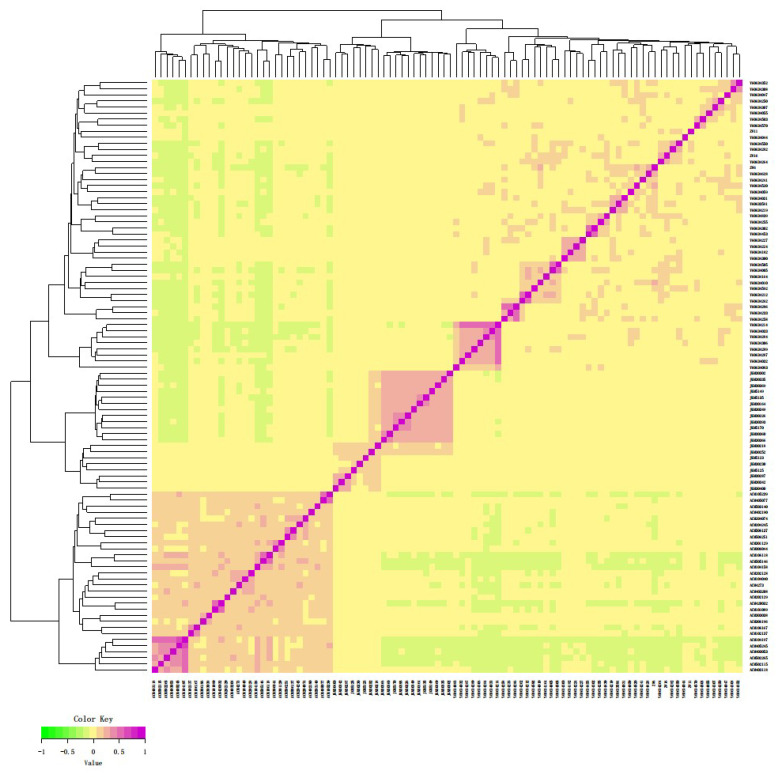
Visualization results of G-matrix analysis. Each small square represents the genetic relationship between two samples. Samples, closer to the red color grid, the larger the value, indicates the closer genetic relationship between two individuals. ACM, Arbas Cashmere male; JGM, Jining Grey male; ZWM, Zhongwei male.

The results of the genomic relationship matrix (G) are shown in Figure 1 wherein the individuals having a close relationship are clustered together and are indicated by violet color.

Individuals of each breed clustered separately where more individuals with a close relationship in the ACM goats, followed by the JGM goats, and relatively few individuals with a close relationship in the ZWM goats. The closely related individuals of ACM goat formed 12 different families. Ten families were found among the individuals of JGM breed. The ZWM goats were genetically divergent from others due to their different pedigrees and the closely related individuals of ZWM goat clustered into 17 separate groups (Figure 1).

**Figure 2:**
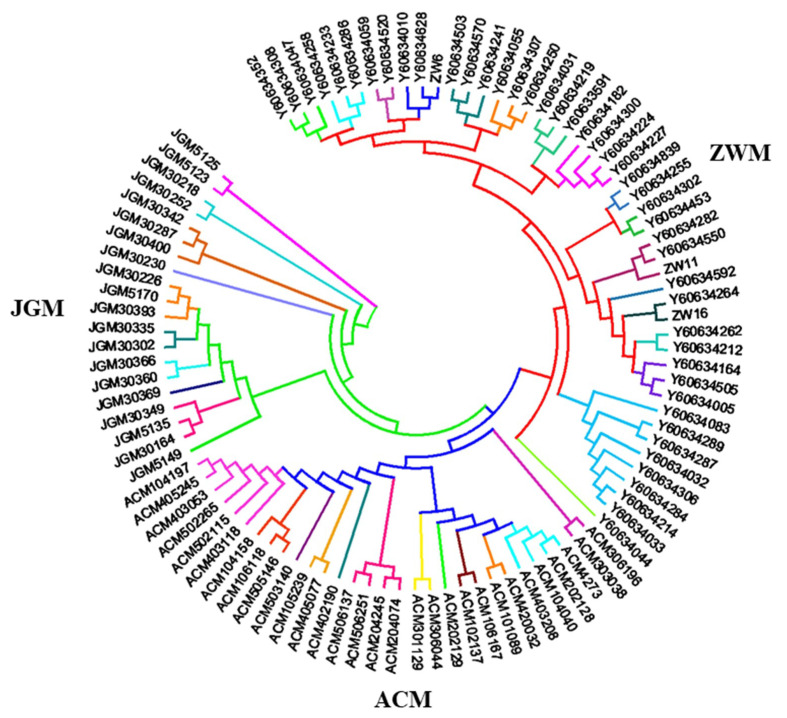
Neighbor-joining (NJ) tree showing different clusters of the studied goat populations. Samples with the same color label are estimated to be samples of the same family in cluster analysis.

A neighbor-joining (NJ) tree revealed that the individuals of studied goat populations are clustered into three major groups where the individuals of each breed formed separate groups according to their genetic relatedness. The individuals marked with the same color in the NJ tree belong to the same family. Therefore, from the cluster analysis, it can be seen that the individuals of ACM, JGM and ZWM breeds separated into 12, 10 and 17 independent sub-groups (Figure 2, Table S1) which is consistent with G-matrix analysis (according to their close genetic relatedness).

**Figure 3:**
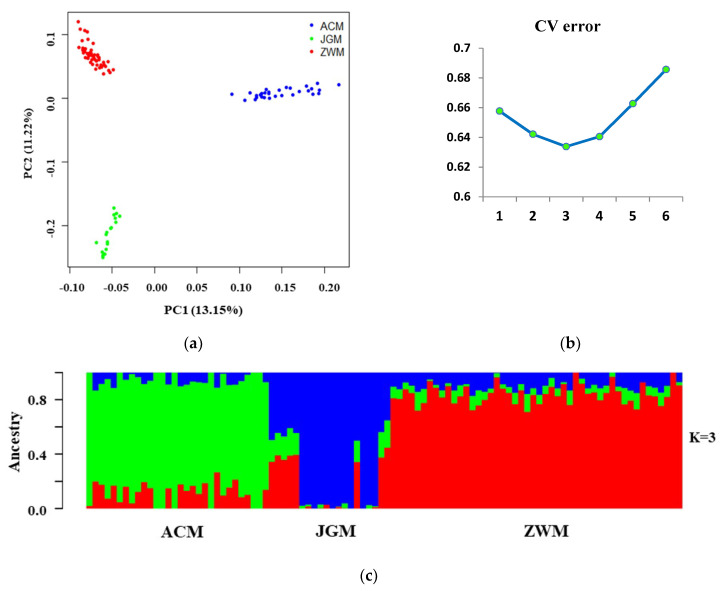
(**a**) Principle component analysis (PCA), (**b**) cross-validation error, (**c**) population structure plot of three goat breeds at K = 3.

The PCA results showed three distinct clusters that were grouped according to the origin and geographical distribution of the studied goat populations (Figure 3a). The first two principal components (PC1 and PC2) explained 24.37%, where JGM breed expresses high levels of genetic variation. Individuals from the ACM breed formed a distinct cluster in a greater distance from the other two. The genetic structure of the population was investigated through Bayesian clustering, assuming a K-value from 1 to 6. Cross-validation (CV) error was the lowest at K = 3, indicating the most likely number of different breeds represented in the 98 samples. At K = 3, three distinct clusters were formed, one for each breed. The ACM samples formed relatively looser clusters, each one enclosing cores of admixture individuals. The genetic background (shown in red in Figure 3c) that predominates in ZWM goats was also present in the ACM breeds.

### 3.3. Genetic Diversity within Breeds

The results of polymorphic SNPs (*P_N_*) and inbreeding coefficient are summarized in Table 3. The proportion of polymorphic SNPs (*P_N_*) was comparable among breeds where the highest value was observed in JGM (96.2%) and the lowest value was found in ACM (91.8%). The JGM goats presented the highest values and ACM displayed the lowest value for both *H_O_* (JGM = 0.401 ± 0.20, ACM = 0.367 ± 0.19) and *H_E_* (JGM = 0.391 ± 0.20, ACM = 0.384 ± 0.20). The ACM exhibited the highest average values for both inbreeding coefficient estimators (*F_ROH_* = 0.086, *F_HOM_* = 0.074) whereas the ZWM showed the lowest *F_ROH_* (0.017). A negative value for *F_HOM_* was observed only for JGM breed (*F_HOM_* = −0.010). A high correlation coefficient between *F_ROH_* and *F_HOM_* was noted for all the breeds ranging from 0.92 (ZWM) to 0.99 (ACM) with an overall mean of 0.92.

**Figure 4:**
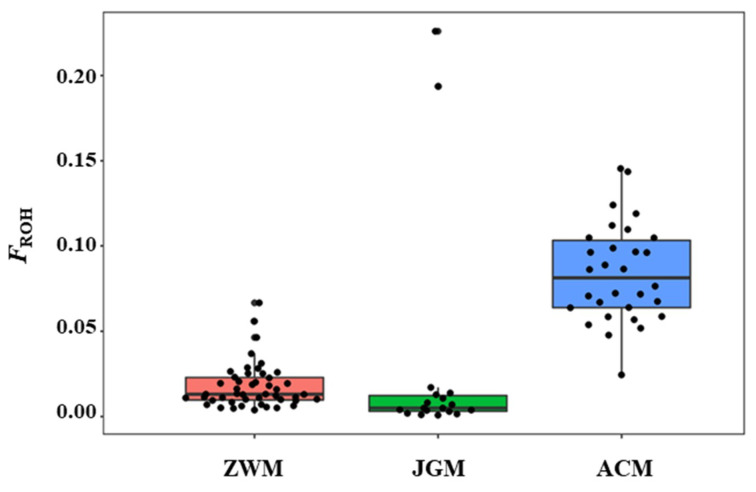
Distribution of *F_ROH_* across breeds.

At the individual level, the individuals of the ACM breed displayed the highest *F_ROH_*, whereas the individuals of JGM breed presented the lowest *F_ROH_* including some individuals with extreme values of *F_ROH_* (Figure 4).

Table 4 illustrates the distribution of *F_ROH_* in different ROH length classes. The ACM goat dominated in *F_ROH_* percentage in all the length classes with the highest value of 4.03% in the 5–15 Mb category. In the long length category (>30 Mb) the highest percentage of *F_ROH_* was 0.79% presented by ACM goats.

**Figure 5:**
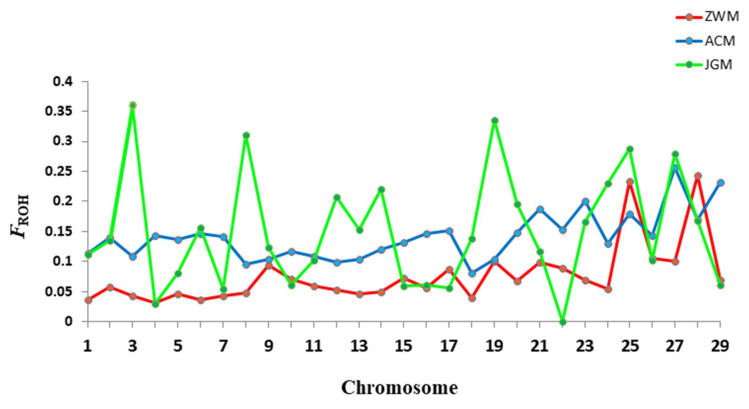
Distribution of inbreeding coefficients (*F_ROH_*) based on runs of homozygosity (ROH) for each chromosome across breeds.

Figure 5 depicts the distribution of inbreeding coefficients (*F_ROH_*) for each chromosome across breeds. In general, the mean *F_ROH_* values followed the same pattern as those computed for the whole genome and differed between chromosomes. We observed that the *F_ROH_* values were highest for chromosome 3, 8, 19 for JGM, chromosome 27, 29 for ACM and for chromosome 25, 28 for ZWM goat breeds.

**Figure 6:**
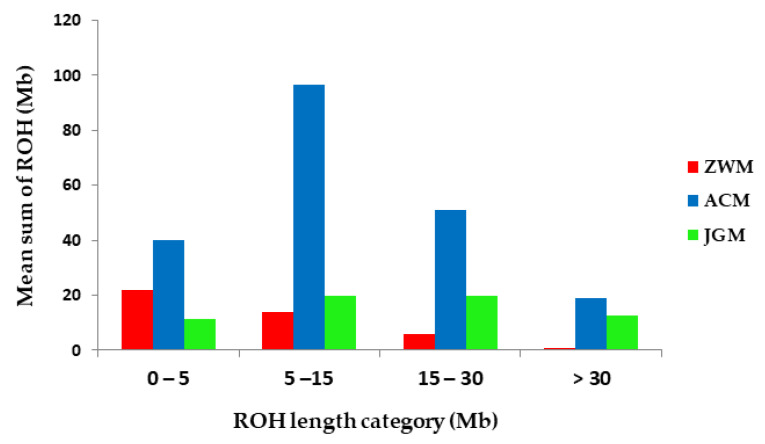
The average sum of runs of homozygosity (ROH) of each breed in different ROH length classes.

To infer the ancient and recent inbreeding, the distribution of the relative numbers of ROHs was plotted in the different length categories within the breeds (Figure 6). The ACM breed displayed the highest mean sum of ROH across the length classes compared to other breeds under study. In the longest length category, the ACM exhibited the highest mean sum of ROH followed by JGM goat breed indicating recent inbreeding in ACM breed.

### 3.4. Effective Population Size

**Figure 7:**
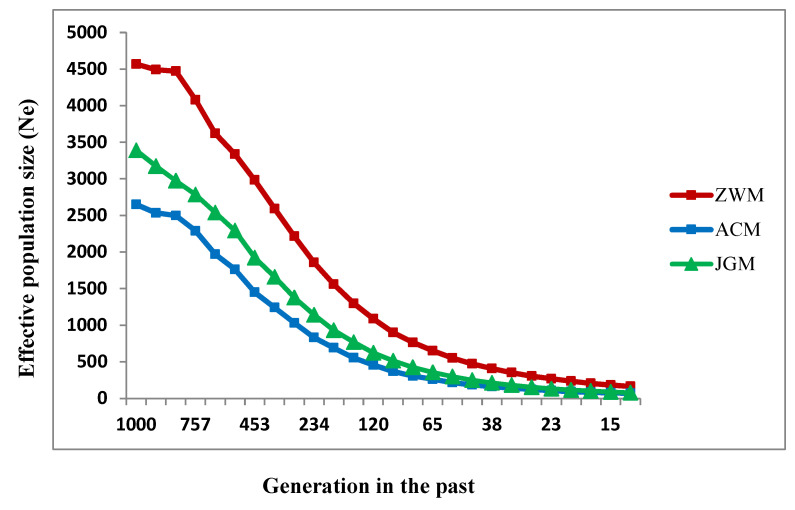
Estimated effective population sizes (*N_e_*) in three Chinese goat populations over the past 1000 generations.

A graphical representation of ancestral and recent effective population sizes (*N_e_*) at different time points for the studied goat populations is presented in Figure 7. The estimated *N_e_* showed a declining trend over time, faster 1000 to 100 generations ago and slower since100 generations across the populations. The most severe drop in current *N_e_* was found in the ACM breed, whereas ZWM presented the highest *N_e_* across the generations (Figure 7). In the three breeds under study, the highest *N_e_* was obtained in ZWM (166), whereas the lowest one was observed in ACM (69) 13 generations ago (Table S2).

## 4. Discussion

Conservation of genetic resources is of central concern globally to ensure sustainable biodiversity and food security. A large number of local animal genetic resources are at risk of extinction due to a lack of conservation policies. Therefore, conservation assessment is essential to design breeding strategies and to manage genetic variability.

The results from the genomic relationship matrix (G) and NJ tree analysis were in agreement, clustering breeds in accordance with their genetic relationship (Figure 1 and Figure 2). The analysis revealed that the individual animal clustered into three major groups, on the basis of the degree of genetic relationship and geographic separation. The analysis confirmed 17, 12 and 10 families among the individuals of ZWM, ACM and JGM goat breeds, respectively. These results indicated that the individuals in the same cluster may share a common ancestry. The conservation farms should have at least six unrelated groups of animals to ensure genetic variability. Although all the goat farms have more than six unrelated groups of goats, their effective population size followed a decreasing trend. The structure analysis result revealed that the genetic background of ZWM goats was also present in the ACM breeds (Figure 3c) which might be attributed to the presence of gene flow due to close geographic distribution or artificial insemination used to develop the ACM goat breed for cashmere traits [26,27]. The structure result indicated that some of the individuals of the ZWM goat breed did not differentiate completely which could be due to the recent establishment of these individuals or high gene flow in this breeds. The close genetic composition and geographic location indicated that a similar conservation strategy can be applied for ZWM and ACM goat breeds. The overall *P_N_* (96.8%) observed in this study indicated that most of the SNPs have been segregating in the breeds under investigation which was comparable to those reported in Pakistani goats [28] and in African goat breeds [29]. More than 95% of the SNPs presented polymorphisms for ZWM and JGM goats, whereas, the proportion of polymorphic loci for ACM goat was 91.8% (Table 3), which could be explained by a relatively higher level of inbreeding in this breed. A comparatively lower level of *P_N_* in ACM goat may reflect its uniqueness and narrow genetic base [28]. Although the studied Chinese goat population displayed a large proportion of informative SNPs, the genetic diversity may be still underestimated because the SNP discovery and development of goat 50K Illumina Beadchip only used African (Boer, Savanna), European (Alpine, Saanen), and a single Asian (Malaysian Katjang) breeds [30]. The lowest *H_O_* was found in ACM (0.367 ± 0.19) goats and the highest in JGM (0.401 ± 0.20), indicating higher genetic diversity existed in JGM goats. The observed heterozygosity reported in this study was lower than the expected heterozygosity which might be due to the Wahlund effect rather than inbreeding. The JGM goats can be proposed as important goat breed for conservation due to unique genetic composition and high level of genetic diversity.

One of the objectives of this study was to evaluate the inbreeding level in the studied goat populations conserved in the state goat farms. The *F_ROH_* coefficients recorded in ZWM goats were lower than that of ACM and JGM goats which might be attributed to the absence of strong selection pressure, high gene flow, and larger effective population size resulted from broad and multiple breeding objective [29]. The ACM exhibited the highest average values for both inbreeding coefficient estimators (*F_ROH_* = 0.086, *F_HOM_* = 0.074) (Table 3). The analysis of *F_ROH_* in different cut-offs showed a high percentage of *F_ROH_* in ACM goats in all the categories. The ACM goats are among the most well-known Cashmere goat breeds and have experienced strong artificial selection for the production of a high Cashmere yield [26]. Attention should be put on this breed to reduce the inbreeding to an acceptable level in the sense that it will not show substantial detrimental effects and to avoid the risk of extinction. The low genomic inbreeding level reported in this study is in agreement with the findings in Ugandan goats [10] and Swiss goat breeds [31]. Strong correlations (r = 0.97) existed between the *F_ROH_* and *F_HOM_* suggesting that the extent of a genome under ROH may be used to infer the aspects of recent population history in the absence of an animal’s pedigree data [32]. The distribution of ROHs coverage was used to infer the recent and ancestral inbreeding. The ACM goats demonstrated a relatively high genomic coverage of very long ROH (>30 Mb), a clear indication that this breed has been subjected to demographic decline and recent inbreeding (Figure 6). Thereby, we need genomics to determine the effective population sizes.

The key advantage of genomic inbreeding coefficients estimation is the availability of chromosomal inbreeding coefficients. The chromosomal patterns of the inbreeding coefficient highlighted the specific effect of selection on these chromosomes for specific traits for which the goats have been selected. The chromosomal patterns of the inbreeding coefficient (*F_ROH_*) in this study were consistent with those reported in Valle del Belice sheep [33] and Danish cattle breeds [4]. The chromosomal *F_ROH_* varied between chromosomes with the highest values obtained for chromosome 3, 8, 19 in JGM, chromosome 27, 29 in ACM and for chromosome 25, 28 in ZWM goat breeds (Figure 5). These might be due to a relatively lower rate of local recombination than the average, which results in high levels of homozygosity on average.

Population size is extremely important in evaluating conservation priorities for a species. The effective population size helps to quantify how a particular population will be affected by genetic drift or inbreeding which could endanger the long term viability of populations. A large population size is generally not a problem, but when the population size becomes small, there will be a greater chance of mating between close relatives, and with those relatives that are likely to carry the same recessive deleterious alleles. Thus, the mating between relatives may lead to inbreeding depression. In this study, we observed an initial pattern of declining *N_e_* with values of 4570, 2652 and 3390 for ZWM, ACM and JGM, respectively, estimated for the past 1000 generations (Figure 6, Table S2). The *N_e_* estimates 13 generations ago found 166 for ZWM goat, suggesting higher genetic diversity and probable high gene flow in this goat farm as supported by a low inbreeding coefficient. Comparatively, a declining current *N_e_* was observed for the ACM and JGM breeds, with their estimated values of 69 and 79, respectively 13 generations ago (Table S2), indicating that these breeds were strongly affected by selection pressure or genetic drift which resulted in population decline. Similar estimates were attained for French and South African goat populations 10 generations ago [34]. A recent study suggested that a current critical effective population size of 100 would be necessary for sustainable animal production in terms of genetic diversity [35,36]. The current effective population size reported in this study is above the threshold only for ZWM breeds, indicating that an initiative is needed in this regard for ACM and JGM goats to maintain sufficiently large current effective population size and consequently to ensure a reasonable diversity for sustainable goat production in the state goat farms. Controls of cross-breeding, development of breeding stations, recording system improvements are key strategies to conserve these breeds under the in situ conservation system. Pastoral production systems have more economic benefits that can affect the conservation of Chinese goats, but recent drought conditions resulting from climate change have restricted this system of production. Moreover, ex situ conservation by gene banks can also be used to preserve genetic diversity as insurance for the future. The optimal contribution of selection can be used in prioritizing the animals to be incorporated in the gene bank. Globalization of breeding programs may help to maintain the genetic diversity and to conserve these local goat genetic resources.

## 5. Conclusions

In this study, we have investigated the pedigree background, level of inbreeding and effective population size in three goat breeds from three state goat farms using the goat SNP 50K BeadChip panel. Genomic relationship and phylogenetic analysis revealed all the populations have more than six groups of animals. High levels of inbreeding were obtained in ACM goat farm, whereas low levels of inbreeding were observed in ZWM and JGM goat farms. High *F_ROH_* values, extremely high genomic coverage of very long ROH (>30 Mb) and severe decline in effective population size indicated strong selection pressure in the Arbas cashmere male goat farm. Thus, initiatives for sustainable conservation and utilization are imperative. The conservation farms should also be aware to maintain sufficiently large *N_e_* for ACM and JGM goats for maintaining genetic diversity. Information from this study may also assist in the reduction of unnecessary inbreeding depression or gene flow among populations in these goat farms. Further studies will be helpful to confirm and refine our results through the inclusion of more samples and other tools such as whole genome re-sequencing, high-density SNP genotyping and phenotypic data.

## Figures and Tables

**Table 1:** Breed name, sample size, location, utility and demography, breeding practices, agro-ecology and raising methods of studied goat populations.

Breed	Sample Size	Location	Utility, Demography and Breeding Practices	Agro-Ecology and Raising Methods	Pop. Trend
Zhongwei male (ZWM)	48	Zhongwei City, Ningxia Hui Autonomous Region	The Chinese Zhongwei goat is used primarily for the production of kid pelts. Total population was 30,000 in 2006. It is an excellent local variety after natural and artificial selection.	Av. Altitude 1200 m. Semi-pasture land, semi-dry land, mainly stall-feeding reared.	Negative
Jining Grey male (JGM)	20	Jining City, Shandong Province, China	They are fast maturing and very prolific, with an average kidding rate of 283%. Total population was 430,000 in 2006. Bred in the local natural environment, after many years of selection their adaptability is high, high fecundity rate.	Av. Altitude 45 m. Agricultural land, wetland, grazing in mountain area in summer and stall feeding in winter	Stable
Arbas Cashmere male (ACM)	30	Erdos City, Inner Mongolia Autonomous Region, China	It is well known for its excellent quality cashmere fiber. Total population was 4.3 million in 2006. After long-term natural and artificial selection under difficult ecological conditions, the cashmere and meat varieties are adapted to the local conditions.	Av. Altitude 1500 m. Semi-pasture land, semi-dry land. Mainly grazing practices.	Positive

**Table 2:** Single nucleotide polymorphisms (SNPs) filtration result.

Parameters	Excluded SNPs	SNP Remained
Total number of SNPs		53,347
SNPs removed due to unrecognized position	2773	50,574
SNP Call frequency (call rate) (<0.95)	1956	48,618
Minor allele frequency (<0.05)	3425	45,193
Hardy-Weinberg equilibrium (*p* < 1 × 10^−5^)	152	45,041
Total SNPs	8306	45,041

**Table 3:** Breed name, sample size (*n*), proportion of polymorphic SNPs (*P_N_*), inbreeding coefficient based on ROH (*F_ROH_*) and based on observed and expected heterozygosity differences (*F_HOM_*), correlation between *F_ROH_* and *F_HOM_*. ACM, Arbas Cashmere male; JGM, Jining grey male; ZWM, Zhongwei male.

Breed	*n*	% *P_N_*	*H_O_*	*H_E_*	*F_ROH_*	*F_HOM_*	*R* (*F_ROH_*, *F_HOM_*)
ZWM	48	95.2	0.385 ± 0.20	0.386 ± 0.19	0.017	0.021	0.92
ACM	30	91.8	0.367 ± 0.19	0.384 ± 0.20	0.086	0.074	0.99
JGM	20	96.2	0.401 ± 0.20	0.391 ± 0.20	0.028	−0.010	0.96
Overall	98	96.8	0.385 ± 0.19	0.397 ± 0.20	0.040	0.032	0.92

**Table 4:** Average percentage genomic inbreeding coefficient (*F_ROH_*) for different length categories of ROH across three goat populations.

Breed	0–5 Mb	5–15 Mb	15–30 Mb	>30 Mb	Total
ZWM	0.92	0.59	0.25	0.03	1.79
ACM	1.67	4.03	2.13	0.79	8.62
JGM	0.47	0.83	0.83	0.53	2.66

## Supplementary Materials

The following are available online at https://www.mdpi.com/2073-4425/11/6/652/s1. Table S1: Pedigree classification of the studied goat populations; Table S2: Effective Population size since 1000 to 13 generations ago.
