# Supplementary material for: Conservation Assessment of the State Goat Farms by Using SNP Genotyping Data
Source: Genes (Basel). 2020 Jun 13;11(6):652. doi: 10.3390/genes11060652 (PMC7349881; doi:10.3390/genes11060652)
Supplement: Supplementary file 1 [file genes-11-00652-s001.zip › Supplementary file/Effective Population size since 1000 to 13 generation ago.docx]

Table S2: Effective Population size since 1000 to 13 generation ago

| GenAgo | ZWM | ACM | JGM |
| --- | --- | --- | --- |
| 1000 | 4570 | 2652 | 3390 |
| 914 | 4493 | 2535 | 3173 |
| 845 | 4473 | 2500 | 2975 |
| 757 | 4080 | 2291 | 2785 |
| 658 | 3622 | 1970 | 2538 |
| 553 | 3339 | 1763 | 2294 |
| 453 | 2986 | 1451 | 1923 |
| 367 | 2592 | 1243 | 1659 |
| 293 | 2217 | 1033 | 1377 |
| 234 | 1857 | 831 | 1143 |
| 187 | 1562 | 691 | 932 |
| 150 | 1297 | 557 | 768 |
| 120 | 1088 | 456 | 623 |
| 98 | 903 | 370 | 515 |
| 80 | 766 | 308 | 426 |
| 65 | 652 | 262 | 355 |
| 54 | 551 | 218 | 301 |
| 45 | 473 | 187 | 249 |
| 38 | 409 | 162 | 213 |
| 32 | 352 | 139 | 182 |
| 27 | 308 | 122 | 155 |
| 23 | 270 | 106 | 135 |
| 20 | 238 | 95 | 118 |
| 17 | 208 | 85 | 102 |
| 15 | 184 | 76 | 90 |
| 13 | 166 | 69 | 79 |
