# Supplementary material for: Conservation Assessment of the State Goat Farms by Using SNP Genotyping Data
Source: Genes (Basel). 2020 Jun 13;11(6):652. doi: 10.3390/genes11060652 (PMC7349881; doi:10.3390/genes11060652)
Supplement: Supplementary file 1 [file genes-11-00652-s001.zip › Supplementary file/Pedigree classification of the studied goat populations.docx]

Table S1: Pedigree classification of Zhongwei male goat

| Family A | Y60634352 | Y60634308 | Y60634047 |  |  |
| --- | --- | --- | --- | --- | --- |
| Family B | Y60634258 | Y60634233 | Y60634286 |  |  |
| Family C | Y60634059 | Y60634520 |  |  |  |
| Family D | Y60634010 | Y60634628 | ZW6 |  |  |
| Family E | Y60634503 | Y60634570 | Y60634241 |  |  |
| Family F | Y60634055 | Y60634307 | Y60634250 |  |  |
| Family G | Y60634031 | Y60634219 | Y60633591 |  |  |
| Family H | Y60634182 | Y60634300 | Y60634224 | Y60634227 |  |
| Family I | Y60634839 | Y60634255 |  |  |  |
| Family J | Y60634302 | Y60634453 |  |  |  |
| Family K | Y60634282 | Y60634550 | ZW11 |  |  |
| Family L | Y60634592 |  |  |  |  |
| Family M | Y60634264 | ZW16 |  |  |  |
| Family N | Y60634262 | Y60634212 |  |  |  |
| Family O | Y60634164 | Y60634505 | Y60634005 |  |  |
| Family P | Y60634083  Y60634284 | Y60634289  Y60634214 | Y60634287  Y60634033 | Y60634032 | Y60634306 |
| Family Q | Y60634044 |  |  |  |  |

Pedigree classification of Arbas cashmere male goat

| Family A | ACM306196 | ACM303038 |  |  |  |
| --- | --- | --- | --- | --- | --- |
| Family B | ACM4273 | ACM202128 | ACM104040 | ACM403208 |  |
| Family C | ACM420032 | ACM101089 |  |  |  |
| Family D | ACM106167 | ACM102137 |  |  |  |
| Family E | ACM202129 |  |  |  |  |
| Family F | ACM306044 | ACM301129 |  |  |  |
| Family G | ACM204074 | ACM204245 | ACM506251 | ACM506137 |  |
| Family H | ACM402190 |  |  |  |  |
| Family I | ACM405077 | ACM105239 |  |  |  |
| Family J | ACM503140 |  |  |  |  |
| Family K | ACM505146 | ACM106118 | 104158 |  |  |
| Family L | ACM403118  ACM104197 | ACM502115 | ACM502265 | ACM403053 | ACM405245 |

Pedigree classification of Jinning grey male goat

| Family A | JGM5125 | JGM5123 |  |  |  |
| --- | --- | --- | --- | --- | --- |
| Family B | JGM30218 | JGM30258 |  |  |  |
| Family C | JGM30342 | JGM30287 | JGM30400 |  |  |
| Family D | JGM30230 |  |  |  |  |
| Family E | JGM30226 | JGM5170 | JGM30393 |  |  |
| Family F | JGM30335 | JGM30302 |  |  |  |
| Family G | JGM30366 | JGM30360 |  |  |  |
| Family H | JGM30369 |  |  |  |  |
| Family I | JGM30349 | JGM5135 | JGM30164 |  |  |
| Family J | JGM5149 |  |  |  |  |
